# On Aqua-Based Silica (SiO_2_–Water) Nanocoolant: Convective Thermal Potential and Experimental Precision Evaluation in Aluminum Tube Radiator

**DOI:** 10.3390/nano10091736

**Published:** 2020-09-01

**Authors:** Tayyab Raza Shah, Hafiz Muhammad Ali, Muhammad Mansoor Janjua

**Affiliations:** 1 Mechanical Engineering Department, University of Engineering and Technology, Taxila 47050, Pakistan; tayyabrazahashmi@gmail.com; 2 Mechanical Engineering Department, King Fahd University of Petroleum & Minerals (KFUPM), Dhahran 31261, Saudi Arabia; 3 Department of Mechatronics and Mechanical Engineering, Higher Colleges of Technology, Dubai P.O. Box 15825, UAE; janjua@gmail.com

**Keywords:** nanofluids, nanotechnology, automotive engines, radiators, aluminum tubes

## Abstract

Although the research on potential use of nanofluids in automotive vehicles is in its embryonic stage, a number of studies have suggested the strong prospect of nanofluids for the efficient thermal management of automotive vehicles. Nevertheless, the pinnacle of nanofluid-based systems awaits stable nanoparticle suspension. The present work studies the heat transfer performance of an aluminum tube automotive radiator with 31 flattened tubes and louvered fins using water and different concentrations (0.04, 0.08, and 0.12 vol.%)-based SiO_2_/water nanofluids as the engine coolant. Inlet temperature and flowrate of the fluid were varied from 60 to 70 °C and 12 to 18 LPM, respectively. The topmost increment in heat transfer rate of 36.92% and Nusselt number of 45.53% were observed in the upper range of tested operational parameters, however, the relative heat transfer increment percentage dropped from 5% (between 0.04 and 0.08 vol.%) to 3.5% (between 0.08 and 0.12 vol.%) due to agglomeration and cluster formation caused by the presence of a greater number of nanoparticles. Precise evaluation of the experimental results was also carried out by reperforming the tests after three days of initial experimentations. A mere deviation of less than 1% was observed between the initial and repeated tests, however, the decline was caused due to the synergistic effects of clustering and fouling.

## 1. Introduction

Nanofluids—being presumed as potential Thermofluids—have been tested in multifarious systems and have evinced exceedingly encouraging results. For the past two decades, appraisal of the potential of nanofluids is underway. Systems in which nanofluids have delineated great potential include photovoltaic thermal systems, electronic cooling, machine cutting, solar energy storage, and radiators. Shah and Ali [1] thoroughly reviewed the use of nanofluids in solar energy applications along with critical analysis of the challenges and practical limitations associated with these systems. They proposed the use of integrated mechanical homogenizers to desist the agglomeration of nanoparticles. Ali et al. [2] presented a brief review of using nanofluids in thermal management of photovoltaic modules for efficient photoelectric conversion and photothermal efficiency maximization. Wahab et al. [3], in a separate review study, analyzed the potential of various nanofluids for solar energy systems. They also underlined similar findings as were presented by Shah and Ali [1].

Ali et al. [4], in another review study, analyzed the preparation of TiO_2_-based nanofluids for various application systems and discussed the performance deterring factors. They underlined pressure drop and sedimentation to be the most crucial challenging factors associated with TiO_2_ nanofluids. Sajid and Ali [5] reviewed the use of nanofluids in a variety of heat exchangers and summarized the associated hurdles. They presented a novel idea to use alike nanocomposites as filler in bounding surface of channels to repel the nano additives inside the nanofluid and desist deposition and consequent fouling in the heat exchanger tubes. Babar et al. [6] presented an interesting review of the preparation, characterization, and applications of modern nanofluids called hybrid nanofluids. All the aforementioned studies agree on the usefulness of nanofluids in energy and thermal management applications. Howbeit, the sole subject of concern associated with nanofluids is their exiguous stability. Different nanofluids have been reported to have different stability periods. The success of nanofluids is entirely dependent on the successful preparation of stable nanofluids that could abort the obstacle of early sedimentation and last for some substantial period of time. Many efforts are underway by the research community in this regard. Several researchers have appraised the stability of nanofluids considering different perspectives, i.e., influential factors. Based on a thorough review of the literature, the critical outlined factors that influence the suspension stability include: concentration of nanoparticles; temperature; method of preparation (single step or two step); power, frequency, and period of ultrasonication; type of basefluid (water, ethylene glycol, oil, etc.); type of nanoparticles. Besides, going further into deep analysis, it has been revealed that there are a number of underlying factors inside each of the aforementioned factors, i.e., for a factor like type of nanoparticle, the underlying factors are the morphology (size, shape, true density, surface area, etc.) and class of nanoparticles (metal, metal oxide, organic, etc.). Asadi et al. [7] performed a detailed analysis of the effect of ultrasonication parameters on thermophysical properties and stability of nanofluids and concluded that it is the time and power of sonication that critically influence the stability, rheology, and thermophysical characteristics of nanofluids. Khan and Arasu [8] conducted a detailed analysis on the effect of synthesis and geometry of nanoparticles on the characteristics of nanofluids in their review article. Likewise, Muthoka and Xuelai [9] conducted a circumstantial evaluation of the effect of nanoparticle size and concluded that the ultimate conclusion of the subject is still to be made, since contradictory results have been reported. Koca et al. [10] also performed a review analysis of the effect of nanoparticle size on the viscosity of nanofluids and concluded that the minute change in size of nanoparticles alters the relative viscosity by up to 40%. Smaller nanoparticle size results in higher stability as well as high thermal conductivity due to a greater extent of Brownian motion, resulting in higher heat transfer rate.

Silicon is the second most abundant element on earth after oxygen (the most abundant element) [11]. Silicon carries exceedingly rich characteristics, owing to which it has found many applications in variety of fields. Silicon has entirely changed the dynamics of electronics and communication industries. Silicon chips have completely revolutionized the engineering fields like mass communication, space science, aviation industry, etc. Another major example of a silicon using field is photovoltaics. Photovoltaic panels made up of silicon are expected to take charge of the power industry in the coming few years as they are yet the most efficient mean of renewable energy generation. Silicon oxide (silica) is broadly used in energy and heat transfer applications and it is finding excessive use as a nano additive. Rahman and Padavettan [12] presented a detailed review of the potential applications of silica nano additives and underlined the underlying characteristics that make silica nanoparticles a strong candidate to be used in a variety of systems. Silica nanoparticles portray low polydispersity index, high biocompatibility, low cost, hydrophilic behavior, high surface area, etc. [13]. Potential fields of silica nanoparticles applications include biomedical as an enzyme or DNA carrier, polymers as filler or reinforcement, aerospace, optical imaging etc. Likewise, the field of nanofluids is not the exception, and the possible use of silicon oxide-based nanofluids has been tested for a number of engineering applications, e.g., chemical industry [14], automotive industry [15], photovoltaic thermal management [16], electronic cooling [17], etc. Nanofluids of SiO_2_ nanoparticles have been reported to lift the performance of the aforementioned systems up to a substantial extent. A short review of studies applying SiO_2_-based nanofluids is presented in Table 1. The applications summary (Table 1) highlights the potential aspects of using SiO_2_-based nanofluids and the most advantageous aspects include minimal pressure drop as compared to rest of the nanofluids, high surge in heat transfer rate, very good dispersion/suspension stability, efficient photothermal conversion, and good thermal and rheological characteristics.

Critical analysis of the literature shows that silica-based nanofluids show very little pressure drop and portray relatively good stability (which is the main challenge pertaining to nanofluids). However, elaboration of thermal potential in many of the systems is not conclusive. Studies report contradictory results regarding the thermal performance of silica nanofluids in different thermal systems like PV/T and vehicle cooling systems. Considering the potential and ability of silica nanofluids to stay stable, it is an imperative need to conduct further evaluation of potential of silica nanofluids so as to research some sort of scientific conclusions. Current research is going to evaluate the performance of silica–water nanofluid in an experimental setup simulating an automotive cooling system. Moreover, the repeatability of experiments is also evaluated in the present study. Various nanofluid samples are set to flow through the radiator at different temperatures and flowrates in the current research endeavor.

## 2. Experimental Details

### 2.1. Making of Nanofluid

**Figure 1:**
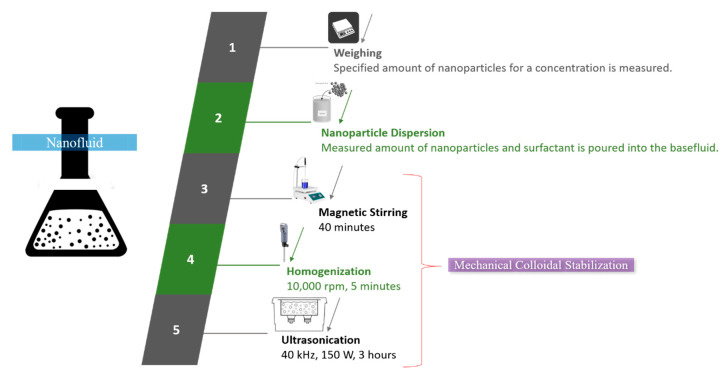
Illustration of SiO_2_–water nanofluid preparation.

**Figure 2:**
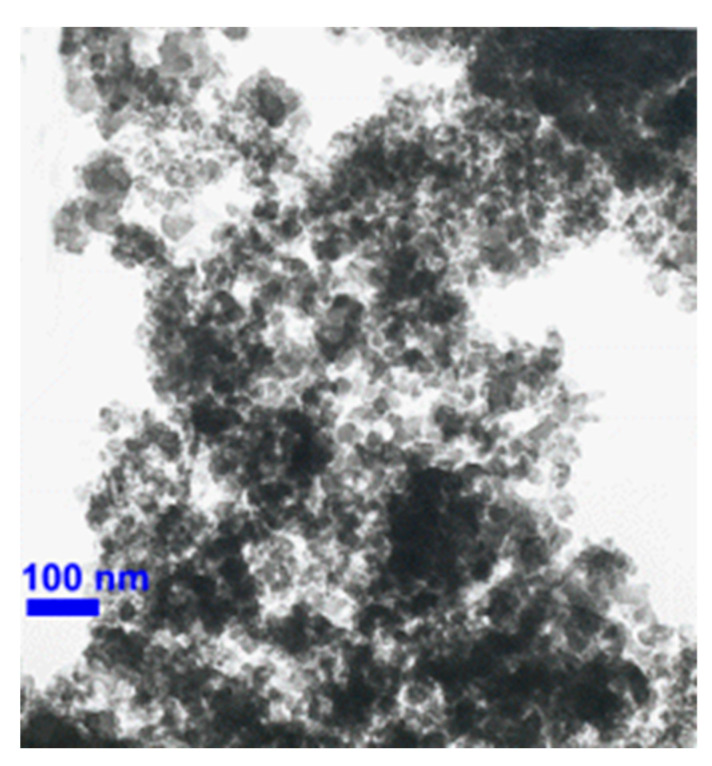
SEM image of SiO_2_ nanoparticles (Courtesy: Nanostructured and Amorphous Materials, Inc., Houston, Pennsylvania, USA).

Colloidal suspension of silica nanoparticles was prepared by adopting a two-step method of nanofluid preparation, as explained in Figure 1. Nanoparticles of silica (SiO_2_) were purchased from NanoAmor (Nanostructured and Amorphous Materials, Inc., Houston, TX, USA). Further description of the key properties of the aforementioned nanoparticles is presented in Table 2. Silica nanoparticles carried white color and spherical morphology, as observed in their SEM image (Figure 2).

**Figure 3:**
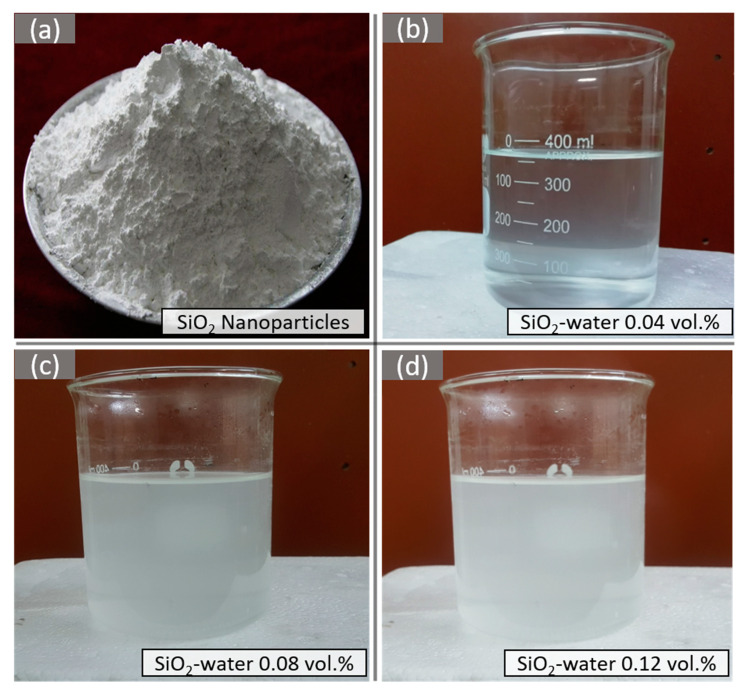
Silica nanoparticles (**a**), SiO_2_–water nanofluid of 0.04 vol.% (**b**), SiO_2_–water nanofluid of 0.08 vol.% (**c**), and SiO_2_–water nanofluid of 0.12 vol.% (**d**).

In order to prepare SiO_2_–water nanofluid, nanoparticles of SiO_2_ (procured from Nanostructured and Amorphous Materials, Inc.) and were poured into water (basefluid) along with a minute amount of CTAB (cetyltrimethylammonium bromide) surfactant. The mixture was stirred in a magnetic stirrer for 40 min at 40 °C and 1100 RPM. Afterwards, the mixture was homogenized in a homogenizer at 10,000 RPM for five minutes and then, was put on ultrasonication (40 kHz and 150 W) for 3 h. Homogenization disseminates the colloidal particles in basefluid and disintegrates the aggregated nanoparticles. Ultrasonication creates stable homogenous colloidal suspension. The prepared nanofluid samples of 0.04, 0.08, and 0.12 vol.% concentrations are shown in Figure 3. Nanofluid samples sustained for more than a month. The formula for computing concentration (vol.%) of nanofluid is presented in Equation (1) [32].

(1) $$\varphi =\left(\frac{\frac{m_{p}}{\rho_{p}}}{\frac{m_{p}}{\rho_{p}}+\;\frac{m_{bf}}{\rho_{bf}}}\right)\;\times 100$$

Thermophysical properties, such as density, heat capacity, thermal conductivity, and viscosity of the prepared nanofluid samples, were evaluated via the correlations that have been used by the researchers previously. Formulas to evaluate the density and heat capacity of the nanofluid samples are presented in Equations (2) and (3), respectively.

(2) $$\rho_{nf}=\;\varphi \rho_{p}+\left(1-\varphi \right)\rho_{bf}$$

(3) $$C_{Pnf}=\frac{\varphi \rho_{p}C_{Pp}+(1-\varphi )\rho_{bf}C_{Pbf}}{\rho_{nf}}$$

Nanofluid’s dynamic viscosity is evaluated by the correlation developed by Corcione [33] presented in Equation (4).

(4) $$\mu_{nf}=\;\mu_{bf}\left[\frac{1}{1-34.87{\left(\frac{d_{p}}{d_{bf}}\right)}^{-0.3}{\left(\varphi \right)}^{1.03}}\right]$$

The parameter “$d_{bf}$” in Equation (4) is the equivalent diameter of the basefluid molecule evaluated by Equation (5) [34].

(5) $$d_{bf}=0.1{\left[\frac{6M}{N\pi \rho_{bf}}\right]}^{1/3}$$

“*M*” and “*N*” in Equation (5) represent the base fluid’s (i.e., water) molecular weight (18.01528 g/mol) and Avogadro’s number (6.022140857 × 10^23^), respectively.

Equation (6), developed by Hamilton and Crosser [35], is used to evaluate thermal conductivity.

(6) $$k_{nf}\;=\;k_{bf}\left[\frac{k_{p}+\left(n-1\right)k_{bf}-\left(n-1\right)\varphi \left(k_{bf}-k_{p}\right)}{k_{p}+\left(n-1\right)k_{bf}+\varphi \left(k_{bf}-k_{p}\right)}\right]$$

“*n*” in Equation (6) represents the shape factor which is 3 for spherical shaped nanoparticles.

### 2.2. Setup Description

**Figure 4:**
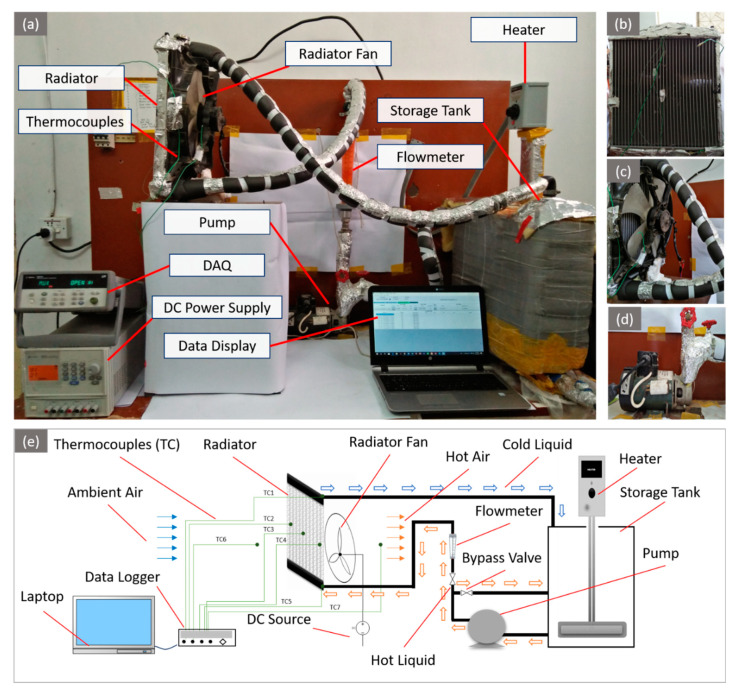
Experimental setup: equipment arrangement (**a**), aluminum tube radiator (**b**), radiator fan (**c**), centrifugal pump (**d**), and schematic illustration (**e**).

The actual experimental setup and a schematic illustration of the experimental setup used for the current research work are presented in Figure 4a–e, respectively. The experimental setup consisted of a liquid storage tank (533.4  × 152.4 × 685.8 mm^3^); a heater (CH-OTS-604, 240 V, and 6 kW, Chromalox, Inc., Pittsburgh, PA, USA) for the sake of elevating the temperature of the water or nanofluid; a pump (for drawing the liquid from the storage tank and moving it through the loop); a flowmeter (rotameter: Omega FL-45100, Omega Engineering, Inc., Norwalk, CT, USA) for measuring the flowrate of the fluid; gate valves for controlling the flow velocity of the liquid; a vehicle radiator along with a forced draft conventional 12 V fan having 700–800 RPM and 7.1 m/s air velocity for effective cooling of the liquid coolant; a DC power supply (Keysight U8032A, 0-60 V, Keysight Technologies, Inc., Santa Rosa, CA, USA) for the radiator fan; thermocouples (K-type Omega 5TC, Omega Engineering, Inc., Norwalk, CT, USA) for recording the temperatures of the liquid, air, and radiator tubes. Values of temperature recorded by the thermocouples were stored in a laptop through a data acquisition system (Agilent 34972-A, Agilent Engineering, Inc., Santa Clara, CA, USA).

Among the setup components, the radiator is the most imperative one. The overall performance of the system is extensively dependent on the radiator’s effectiveness (design of the fins and tubes). A car radiator with louvered fins and 31 aluminum flattened tubes was installed to cool the engine coolants (water and nanofluid). Important details of the radiator are presented in Table 3.

Geometrical calculations of the radiator-like tube and fin specifications are performed by using several formulas. To calculate the cross-sectional area of the radiator tubes, Equation (7) is used.

(7) $$A_{c_{t}}=n\left(W\times T\right)$$

In Equation (7), “*n*” is the number of radiator’s tubes and “*W*” and “*T*” are the width and thickness of the radiator tubes, respectively.

Equation (8) presents the formula to compute the surface area of the tubes.

(8) $$A_{s_{t}}=n\left(2\right)\left(H\times W+\;H\times T\right)$$

Hydraulic diameter of radiator tubes is calculated by Equation (9) [30].

(9) $$D_{h_{t}}=\frac{4\times \left[\frac{\pi }{4}W^{2}+\left(T-W\right)\times W\right]}{\pi \times W+2\times \left(T-W\right)}$$

## 3. Data Reduction

### 3.1. Thermal Performance

Various parameters used to assess the thermal performance of nanofluid include the heat transfer rate, CHTC, Nusselt number, OHTC, LMTD, and overall efficiency of the heat exchanger, i.e., the radiator.

Heat transferred by the engine coolant, i.e., water or nanofluid through the radiator, is calculated by Equation (10).

(10) $$Q_{wf}=m_{wf}C_{P_{wf}}\left(T_{in_{wf}}-T_{out_{wf}}\right)$$

Subscript “*wf*” stands for the engine coolant (either water or nanofluid), whereas “*T_in_*” and “*T_out_*” represent the temperature of engine coolant at the inlet and outlet of the radiator, respectively.

The convection heat transfer rate by the engine coolant is calculated by Equation (11).

(11) $$Q_{c_{wf}}=h_{wf}A_{s_{tube}}\left(T_{b_{wf}}-T_{s_{tube}}\right)$$

In Equation (11), subscript “*t*” represents the tube, and “$T_{b_{f}}$” and “$T_{s_{t}}$” represent the engine coolant’s bulk temperature (average of inlet and outlet temperature through the radiator) and the tube’s surface temperature, respectively. Moreover, CHTC “$h_{f}$” is calculated by Equation (12).

(12) $$h_{f}=\frac{m_{wf}C_{P_{wf}}\left(T_{in_{wf}}-T_{out_{wf}}\right)}{A_{s_{tube}}\left(T_{b_{wf}}-T_{s_{tube}}\right)}$$

The Nusselt number is computed by employing Equation (13).

(13) $$Nu=\frac{h_{wf}D_{h_{tube}}}{k_{wf}}$$

The heat of the engine coolant is removed by the air flowing across the radiator’s outer surface. The amount of heat removed by the air is calculated by Equation (14).

(14) $$Q_{air}=m_{air}C_{P_{air}}\left(T_{out_{air}}-T_{in_{air}}\right)$$

The value of the convective heat transfer coefficient for air is estimated by Equation (15) [36].

(15) $$h_{air}=\frac{m_{wf}C_{P_{wf}}\left(T_{in_{wf}}-T_{out_{wf}}\right)}{A_{s_{rad}}\left(T_{s_{tube}}-T_{b_{air}}\right)}$$

To calculated OHTC, Equation (16) is used.

(16) $$U=\frac{1}{\frac{1}{\eta_{total}h_{air}}+\frac{1}{\left(\frac{A_{s_{tube}}}{A_{s_{rad}}}\right)h_{wf}}}$$

Parameter “$\eta_{total}$” (total heat exchanger efficiency) in Equation (16) is calculated by Equation (17).

(17) $$\eta_{total}=\;\left(\frac{A_{s_{fin}}}{A_{air}}\right)\eta_{fin}+1-\left(\frac{A_{s_{fin}}}{A_{air}}\right)$$

“$A_{s_{fin}}$” is surface area of fins and “*A_air_*” is the area through which the air flows. The parameter “$\eta_{fin}$” (fin efficiency) in Equation (17) is calculated by Equation (18).

(18) $$\eta_{fin}=\frac{tanh\left(mL\right)}{mL}$$

where

(19) $$m=\;\sqrt{\frac{2h_{air}}{k_{fin}t}}$$

“*L*” and “*t*” in Equations (18) and (19) are the length and thickness of fin, respectively.

“*A_a_*” is the total area of the radiator and is calculated by Equation (20). The total area of the radiator is found by adding the surface area of the bare tubes and the surface area of the fins.

(20) $$A_{a}=A_{s_{tube}}+A_{s_{fin}}$$

Some other dimensionless numbers, like the Reynolds number (*Re*) and Prandtl number (*Pr*), are calculated by Equations (21) and (22), respectively.

(21) $$Re=\frac{\rho_{wf}V_{wf}D_{h}{}_{t}}{\mu_{wf}}$$

(22) $$Pr=\frac{\mu_{wf}C_{p_{wf}}}{k_{wf}}$$

### 3.2. Uncertainty Analysis

The uncertainty determination method proposed by Moffat [37] could be used to evaluate the uncertainty of an experimental setup by considering the precision of components stated by the manufacturers. Furthermore, the uncertainty of parameters like temperature, flowrate, heat transfer rate, etc., could be computed by the method devised by Kline and McClintock [38]. Employing the aforementioned methods, Selvam et al. [36] formulated a simple equation for uncertainty determination for the systems like the current one. They formulated the equations to determine the uncertainty in heat transfer rate, CHTC for both air side and liquid side, and OHTC. Uncertainty in heat transfer rate could be calculated by the expression in Equation (23).

(23) $$\delta Q_{wf}=\sqrt{{\left(\frac{\delta Q_{wf}}{\delta m_{wf}}\delta m_{wf}\right)}^{2}+{\left(\frac{\delta Q_{wf}}{\delta C_{P}{}_{wf}}\delta C_{P_{wf}}\right)}^{2}+{\left(\frac{\delta Q_{wf}}{\delta T_{in_{wf}}}\delta T_{\;in_{wf}}\right)}^{2}+{\left(\frac{\delta Q_{wf}}{\delta T_{out_{wf}}}\delta T_{out_{wf}}\right)}^{2}}$$

where

(24) $$\frac{\delta Q_{wf}}{\delta m_{wf}}=C_{P_{wf}}(T_{out_{wf}}-T_{in_{wf}}),\frac{\delta Q_{wf}}{\delta C_{P}{}_{wf}}\;=\;m_{wf}\left(T_{out_{wf}}-T_{in_{wf}}\right),\;\frac{\delta Q_{wf}}{\delta T_{in_{wf}}}=m_{wf}C_{P_{wf}},\frac{\delta Q_{wf}}{\delta T_{out_{wf}}}=m_{wf}C_{P_{wf}}$$

The expressions in Equation (24) involving the sign “δ” refer to uncertainty i.e., “$\delta Q_{f}”$ represents the uncertainty in heat transfer rate, “$\delta m_{f}”$ represents the uncertainty in mass flowrate and so on.

By employing Equation (23), an uncertainty of ±7%, and ±6.7% in heat transfer rate was observed for water and nanofluid, respectively. The aforementioned uncertainties could well be attributed to 2.8% uncertainty in flowrate of the fluid.

## 4. Results and Discussions

### 4.1. Setup Validation

The accuracy of the experimental setup is validated by comparing the experimental results with the results obtained by empirical correlations. The three most extensively used correlations by researchers to validate experimental setups in close proximity to the current setup have been used for the sake of validity evaluation.

The empirical correlation proposed by Dittus and Boelter [39] (Equation (25)) is the most frequently used correlation for the validation evaluation of setups similar to the current one.

(25) $$Nu_{wf}=0.0236Re^{0.8}Pr^{0.3}.$$

Another frequently used empirical correlation is proposed by Gnielinski [40] (Equation (26)). This correlation is applicable for the 2300 − 5 × 10^6^ Reynolds number range and the 0.5–2000 Prandtl number range.

(26) $$Nu_{wf}=\frac{\left(\frac{f}{2}\right)\left(Re_{wf}-1000\right)Pr_{wf}\;}{1+12.7{\left(\frac{f}{2}\right)}^{0.5}(Pr^{\frac{2}{3}}-1)}$$

In Equation (26), “f” is the friction factor which is calculated by the formula presented in Equation (27).

(27) $$f={\left(1.58lnRe_{wf}-3.82\right)}^{-2}$$

The third most significant correlation is presented by Petukhov [41] (Equation (28)), which is applicable for the 3000 − 5 × 10^6^ Reynolds number range and the 0.5–2000 Prandtl number range.

(28) $$Nu_{wf}\;=\;\frac{\left(\frac{f}{8}\right)Re_{wf}Pr_{wf}\;}{1.07\;+\;12.7{\left(\frac{f}{8}\right)}^{0.5}(Pr^{\frac{2}{3}}-1)}$$

where

(29) $$f={\left(1.82logRe_{wf}-1.64\right)}^{-2}$$

**Figure 5:**
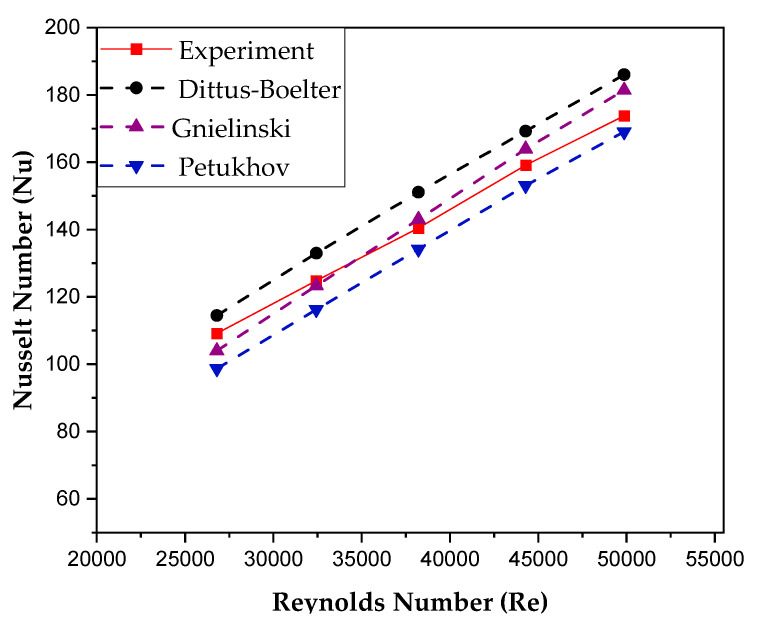
Setup validation.

The Nusselt number variation against the Reynolds number for water (65  °C inlet temperature) is presented in Figure 5. Nu is found to increase with the increase in Re because of the increased convection heat transfer resulting from mounting turbulence.

As is evident in Figure 5, experimental trends are in close proximity to the correlation-based trends. The mean absolute error percentages are 4.70%, 5.99%, and 8.66% between experimental values and Dittus and Boelter correlation, Gnielinski correlation, and Petukhov correlation, respectively. In this way, the authenticity/accuracy of the experimental setup becomes unquestioned.

### 4.2. Heat Transfer

**Figure 6:**
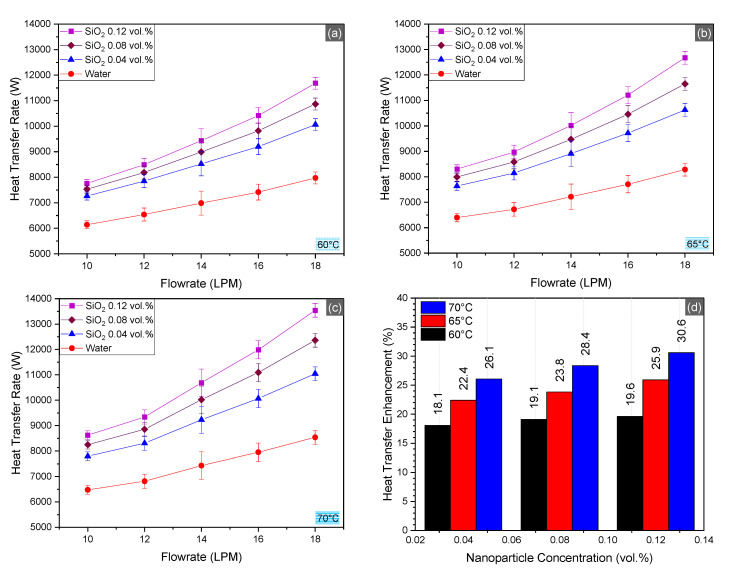
Convection heat transfer in the radiator at inlet temperatures 60–70 °C (**a**–**c**) and average enhancement in heat transfer rate for SiO_2_/water nanofluid as compared to the basefluid (**d**).

The results of the convective heat transfer evaluation tests in the aluminum tube radiator (31 tubes) are drawn in Figure 6. Three nanofluid concentrations (0.004, 0.08, and 0.12 vol.%) were tested at different inlet temperature and flowrate. Increase in inlet temperature, flowrate, and concentration of the fluid increased the rate of heat transfer in the radiator. Nevertheless, the increase in nanofluid concentration and flowrate were found to be the major causes of heat transfer amelioration. Maximum increase in heat transfer rate was observed to be 36.95% as compared to water at 70 °C inlet temperature, 18 LPM flowrate, and 0.12 vol.% of nanoparticles, whereas the maximum increment for 0.08 vol.% was observed at 30.92% and for 0.04 vol.%, the enhancement was 22.71%. Average increase in heat transfer rate was observed to be 19% for 0.04 vol.%, 24% for 0.08 vol.%, and 28% for 0.12 vol.%. Average percentage of relative heat transfer augmentation started to drop from 5% (between 0.04 and 0.08 vol.%) to 3.5% (between 0.08 and 0.12 vol.%). The impact of inlet temperature increment was negligible on the system performance, as only 1% enhancement was observed when the temperature increased from 60 to 65 °C. Abbas et al. [42] reviewed the potential of nanofluids to be used as coolant in radiators and generalized similar trends observed in the current work.

Anomalous increase in heat transfer rate for nanofluid is attributed to the exceptional thermal characteristics induced by the nanoparticle basefluid interface [43]. The presence of nanoparticles in basefluid provides a medium for rapid thermal energy transfer due to the higher thermal conductivity of the particles. Increasing the concentration of nanoparticles provides greater mean of heat transfer within the fluid. Besides, the formation of nanolayers (i.e., solid-like structure of the basefluid around the nanoparticle) is one of the major causes which leads to anomalous heat transfer in the nanofluids [44]. There are various theories like effective medium theory that tend to explain the thermophysical science of nanofluids [45]. Brownian motion of nanoparticles is also a major cause of heat transfer elevation. There are a number of studies that studied the effect of Brownian motion in nanofluids because Brownian motion results in increased nanoparticle collision. However, increased nanoparticle interactions cause agglomeration and subsequent cluster formation, which leads to the sedimentation of nanoparticles since there are instinctive cohesive forces in the nanoparticles. Nevertheless, there are also some theories that describe the ideal suspension of nanoparticles as deleterious for heat transfer in the nanofluids [46].

High temperature of nanofluids poses extreme challenges, as high temperature causes higher Brownian motion, which leads to increased interaction and subsequent agglomeration due to instinctive cohesive and Van der Waals attractive forces. Repeated thermal cycles, i.e., heating and cooling of nanofluids, results in fouling of channels caused by deposition of the nanoparticles over the channel surface. Fouling can block the heat transfer through the channel walls [47]. High temperature also causes the surfactant to wear [48].

### 4.3. Nusselt Number (Nu)

**Figure 7:**
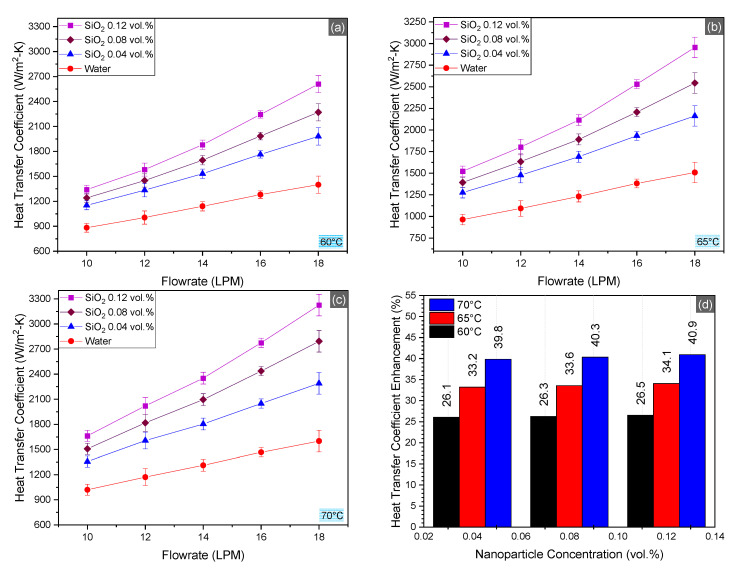
Convection heat transfer coefficient in the radiator at inlet temperatures 60–70 °C (**a**–**c**) and average enhancement in heat transfer coefficient for SiO_2_/water nanofluid as compared to the basefluid (**d**).

Trends of convective heat transfer coefficient, as obtained by the experimental results, are plotted in Figure 7. The topmost enhancement in the convective heat transfer coefficient with respect to the basefluid was observed 57.59% at the upper limit of the tested operational conditionals, i.e., 70 °C inlet temperature, 18 LPM flowrate, and 0.12 vol.% concentration of nanoparticles. The average increase in CHTC was 26.2% for 0.04 vol.%, 33.5% for 0.08 vol.%, and 40% for 0.12 vol.% of nanofluid. The momentous mount in CHTC is attributed to increased surface area for heat transfer in the basefluid [49]. Moreover, turbulence caused by high flowrates also develops immense impact on the convective thermal potential of nanofluids [50,51,52,53,54].

**Figure 8:**
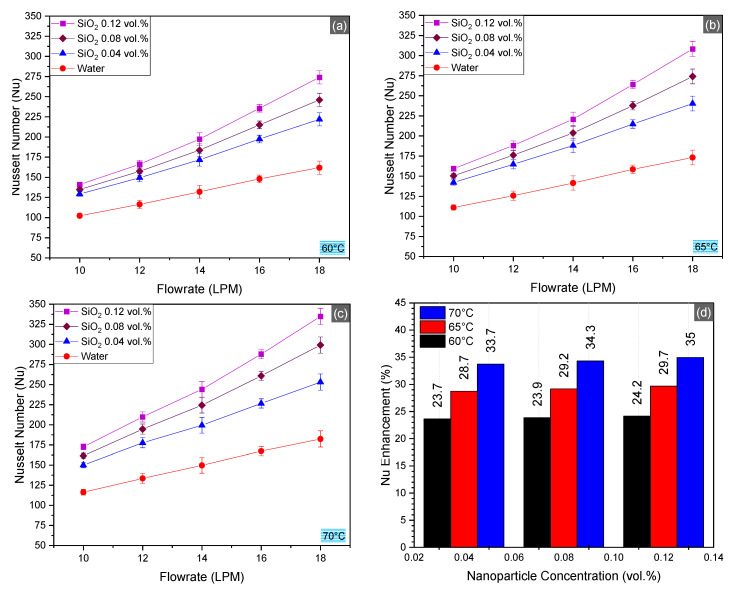
Nusselt number (Nu) in the radiator at inlet temperatures 60–70 °C (**a**–**c**) and average enhancement Nu for SiO_2_/water nanofluid as compared to the basefluid (**d**).

Percentage enhancement in the Nusselt number is reported to be greater than enhancement in heat transfer for SiO_2_/water nanofluid unlike other nanofluids. Increased convective heat transfer leads to Nusselt number elevation in nanofluids. The Nusselt number results are presented in Figure 8. Maximum enhancement of Nusselt number was observed to be 45.53% in the upper range of operational parameters. Average increments were found to be 23.7%, 29.45%, and 34.5% for 0.04, 0.08, and 0.12 vol.%, respectively.

The Nusselt number increment is attributed to locally optimal convective heat transfer characteristics such as convective heat transfer coefficient, surface area of the nanoparticles, highly turbulent flow of the fluid, and nanoparticle concentration [55,56,57]. The performance of nanofluids is extensively influenced by the local conditions of the test. Hussein et al. [30] experimentally evaluated the performance of the SiO_2_/water nanofluid in the radiator and reported 40% maximum enhancement in Nusselt number at 0.4 vol.% concentration of nanoparticles. Hussein et al. [30] reported 56% enhancement in Nusselt number at 2.5 vol.% of SiO_2_/water nanofluid. They suggested to use smaller nanoparticle concentrations to obtain optimal results. Therefore, we tested smaller concentrations of SiO_2_ nanoparticles, and the results depicted an increase of 45.53% at 0.12 vol.%.

### 4.4. Radiator Efficiency

**Figure 9:**
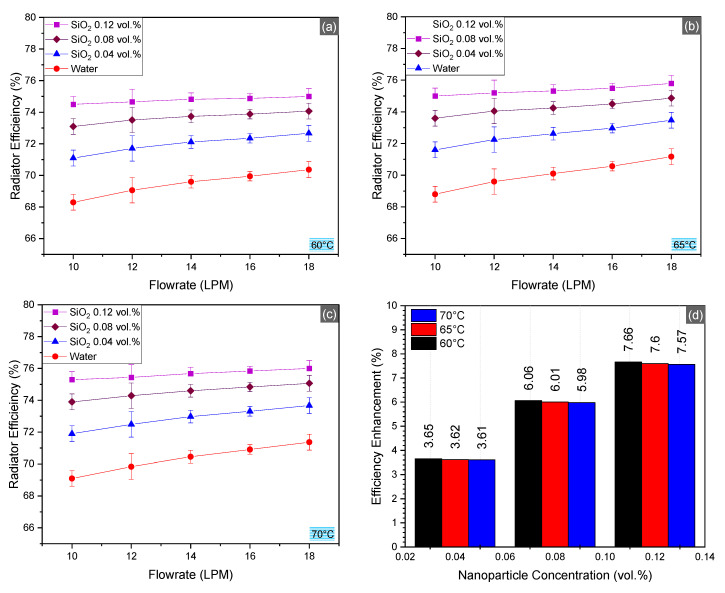
Total efficiency of the radiator at inlet temperatures 60–70 °C (**a**–**c**) and average enhancement in efficiency for SiO_2_/water nanofluid as compared to the basefluid (**d**).

Radiator efficiency calculations revealed that the use of nanofluids results in a minor increment, unlike convective heat transfer. The radiator’s efficiency trends are presented in Figure 9. Maximum efficiency enhancement was obtained as 7.57%. These results could well be understood by considering the fact that the total efficiency of the radiator is more dependent on the air side geometry of the radiator, i.e., radiator fin geometry, fin material, and air flow geometry. For the stated reasons, the total efficiency of the radiator can be increased by modifying/optimizing the air side section of the radiators.

### 4.5. Prediction Model

The Nusselt number is the major parameter to consider while studying the convective thermal potential of a system. Considering the significance of the Nusselt number, a number of studies have proposed correlations or models to predict its values. A large number of past studies have used regression modeling to develop a predictive model of Nusselt number in a heat exchanger using nanofluids [54,55,56,57,58]. Sundar et al. [58] used regression modeling to develop friction factor correlation. Kuznetsov et al. [55] developed a regression model to predict the Nusselt number. Janardana et al. [56] also employed regression modeling to develop a Nusselt number correlation for a CuO/water–propylene glycol nanofluid in heat exchanger tubes with twisted inserts. Jahan et al. [57] presented regression analysis to develop a Nusselt number correlation for convectively heated stretching/shrinking metal sheet. Sundar et al. [58] also employed regression modeling to develop a correlation to predict friction factor inside a plain tube using a Fe_3_O_4_ magnetic nanofluid One such model has also been developed using regression modeling, as presented in Equation (30).

(30) $$\begin{array}{c} Nu_{Reg}=0.299\dot{m^{2}}-4388.126\varphi^{2}-0.042T^{2}-14.147\dot{m}-1656.386\varphi \\ +4.231T+86.379\dot{m\varphi }+25.398\varphi T+0.216\dot{m}T-13.968 \end{array}$$

**Figure 10:**
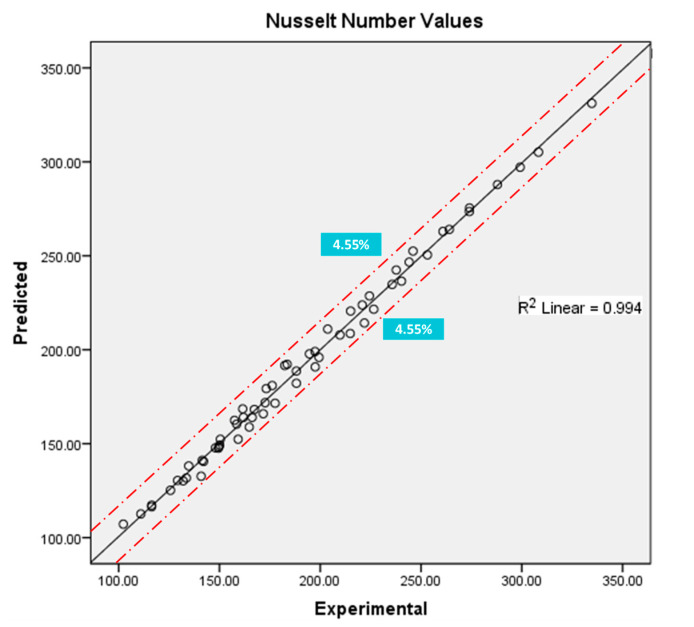
Validation of proposed regression model with the experimental data.

According to the proposed model, the value of the Nusselt number depends on the values of flowrate, nanoparticle concentration, and inlet temperature of the fluid. The proposed model is valid in the range of 10–18 LPM flowrate, 0–0.12 vol.% concentration of SiO_2_ nanoparticles in the basefluid (water), and 60–70 °C inlet temperature. The proposed model fits almost perfectly within the aforementioned range of operational parameters, as Figure 10 of predicted and experimental Nusselt number values depicts. The *R^2^* value of the graph of Nusselt number values of predicted and actual experimental values is 0.994. The margin of deviation was calculated using the formula as presented in Equation (31) [59]. Average margin and highest margin of deviation were found to be 1.845% and ±4.55%.

(31) $$Margin\;of\;deviation\;\left(Dmargin\right)=\;\left[\frac{{\left(Nu\right)}_{Exp}-\;{\left(Nu\right)}_{Pred}}{{\left(Nu\right)}_{Pred}}\right]\;\times 100\;\left(\%\right)$$

### 4.6. Repeatability

**Figure 11:**
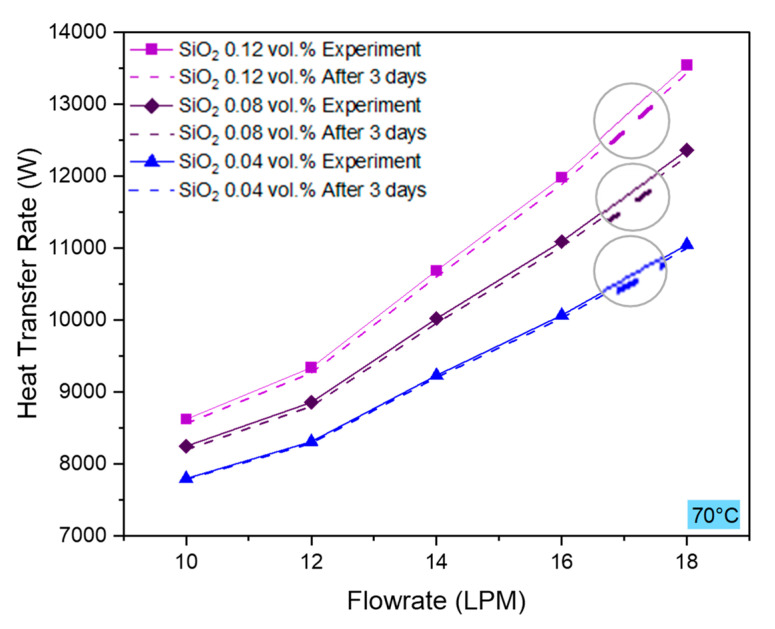
Experimental repeatability test results of the heat transfer rate performed after three days of initial experimentation.

**Figure 12:**
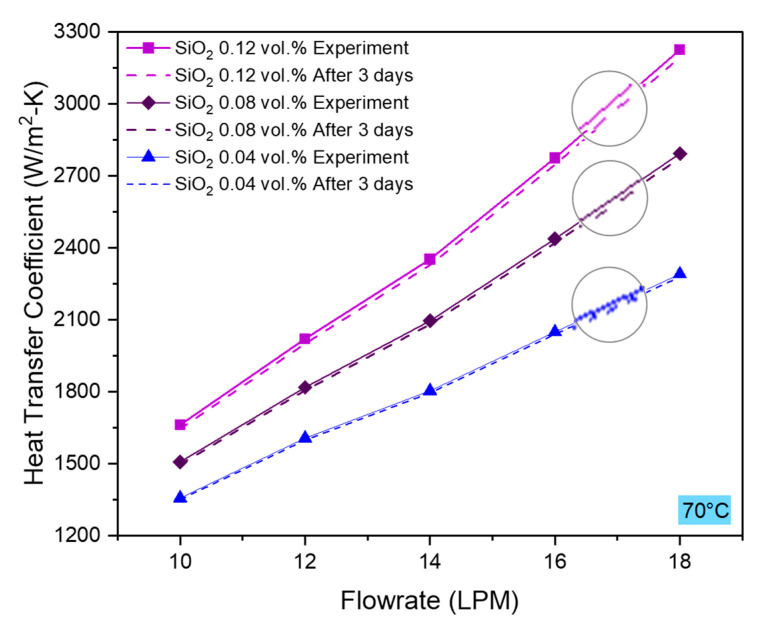
Experimental repeatability test results of the heat transfer coefficient performed after three days of initial experimentation.

To appraise the precision of our experimental findings, we reperformed the experiments after three days of initial experimentation and observed encouraging heat transfer rate and heat transfer coefficient results, as shown in Figure 11 and Figure 12. Repeatability tests were performed for all the concentrations of nanofluids at 70 °C inlet temperature. Though mere (≤1%), the deviation between initial and reperformed tests increased with increasing the concentration because the presence of a larger number of nanoparticles makes it prone to cluster formation. Nevertheless, agglomeration effect can also sometimes increase the thermal performance if significant thermal percolation phenomenon takes place [60]. Furthermore, the heat transfer coefficient test results presented in Figure 11 represent a decline in heat transfer coefficient as well. A drop in the convective heat transfer coefficient could be attributed to the synergistic effects of clustering and fouling. Fouling is primarily caused by surface wear due to surface rubbing and particle deposition on the bounding surface due to thermal cycles. High flowrate and high concentration of nanoparticles can cause greater wear of bounding surface by the nanoparticles, as observed in Figure 12.

**Figure 13:**
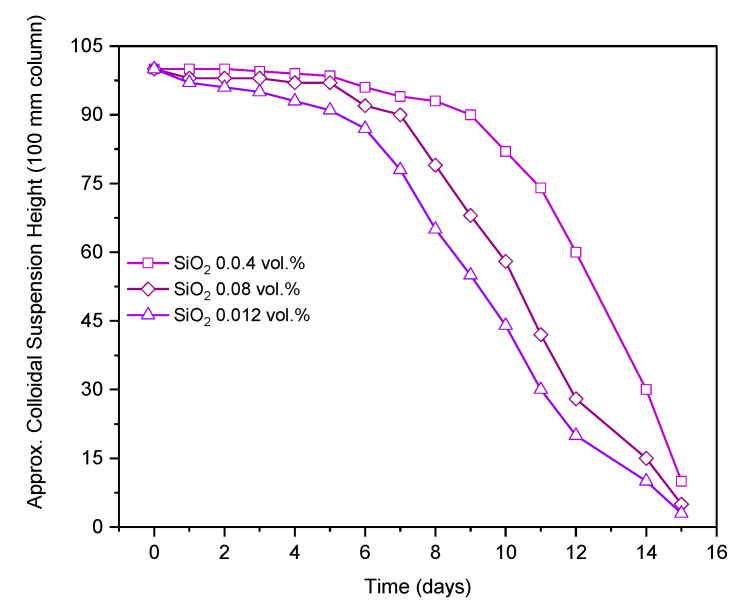
Colloidal suspension height in a column over time.

Nanofluid samples were perfectly stable for six days and complete sedimentation was observed after 11 days The suspension stability of the nanofluid samples was assayed using a visual sedimentation evaluation method, in which the samples were placed in 100 mm columns and the height of colloidal suspension was examined. The approximate height of colloidal suspension over the time is presented in Figure 13. Rate of sedimentation was observed to increase with the passage of time once the sedimentation had started. A rapid decline in suspension height can be observed in Figure 13.

### 4.7. Practical Implications

A number of practical implications can be drawn from the aforesaid experimental findings of the current research work. The obtained findings hold mammoth significance for industrial realization of such automotive cooling systems that could utilize nanofluids as coolants. The most significant takeaways from the current research are that silica-based nanofluids could prove to be a viable option as a replacement of conventional coolants as well as other nanofluids for automotive cooling systems due to high convective thermal potential even at low concentration, prolonged suspension stability, and facile operation control as it displays sustained results over a considerable period of time. Howbeit, the worrisome aspect of these findings is the observed demining of convective heat transfer coefficient due to fouling caused by rubbing and deposition of nano additives over the bounding surface. Moreover, maximum improvements have been observed at higher flowrate of the fluid, which is also a challenging aspect of the findings since it can cause surface erosion and material deterioration due to the high-pressure streams of the fluid. The aluminum-made radiator tubes have a wall thickness of 1 mm and possess moderate strength to withstand such high-pressure streams. Practical systems designed to operate for a longer period of time would be greatly prone to surface deterioration due to the erosion potential of nanofluids. For the stated reasons, it is imperative to use hydrophobic material coating over the bounding surface to desist chemical activity. Finally, the radiator’s efficiency analysis depicted a comparatively minute influence of using nanofluids on total efficiency of the radiator due to its greater dependency on air side geometry, fin geometry, fin material, tube material and tube geometry, air flow velocity, and ambient temperature.

## 5. Conclusions and Future Directives

Experimental precision and convective thermal potential of low concentration SiO_2_/water nanofluid in an aluminum tube automotive radiator has been evaluated in this experimental study. Detailed comparative analysis of using water and nanofluid as coolants in terms of convective heat transfer parameters (i.e., convection heat transfer rate, convection heat transfer coefficient, and Nusselt number) along with scientific interpretation of the obtained results have been presented. A range of operational parameters (inlet temperature, flowrate, and nanoparticle concentration) was tested to analyze the effect of varying the values of aforementioned parameters on convection heat transfer in a flattened tube radiator. Major findings of the current research work and future recommendations are listed as follows.

- Among the tested range of operational parameters (60–70 °C inlet temperature, 12–18 LPM flowrate, and 0.04–0.12 vol.% nanoparticle concentration), the topmost increments in convection heat transfer parameters were observed in the upper range of examined values of working conditions (i.e., 70 °C inlet temperature, 18 LPM flowrate, and 0.12 vol.% nanoparticle concentration). A remarkable enhancement of 36.92% in heat transfer rate and 45.53% in Nusselt number was observed in the upper range of tested operational conditions. Nevertheless, the percentage of increment in heat transfer with respect to preceding concentration of nanoparticles dropped past 0.08 vol.% nanoparticle concentration.
- Discrete comparison of the literature and current findings reveals that the impact of inlet temperature tends to dwindle at higher range due to the fact that at higher temperatures, the colloidal suspensions are more likely to lose suspension uniformity, which leads to heat transfer rate deterioration. Nonetheless, the Reynolds number/flowrate of the nanofluid leaves a massive impact on the thermal performance of the system resulting from the turbulence created in the flow. A slight increment flowrate results in huge heat transfer rate augmentation, however, the upper range of flowrate is retrained by available pumping power.
- Improvement in heat transfer rate or Nusselt number was found to be extensively dependent on the local conditions.
- Lesser influence of the nanofluid’s usage was observed on total efficiency of the radiator due to greater influence of air side section parameters.
- A regression model having a maximum of ±4.55% margin of deviation has been presented in this study to predict the Nusselt number.
- Owing to the sustained suspension uniformity of colloidal nanoparticles obtained by both chemical and mechanical stabilizing processes as well as small size and low concentration of nanoparticles, impressive precision/repeatability of the experimental results of the SiO_2_/water nanofluid was observed with a mere less than 1% deviation.
- Microscale challenges associated with thermal systems using nanofluids similar to the one tested in the current research include the channels’ interior surface degradation due to presence of colloidal particles, clogging in the channels, particle deposition on the contact surface, etc. Repeated thermal cycles can cause fouling in the flow channels by the deposition of nanoparticles on the bounding of the channel.
- There is mere prospect of success of nanofluid-based thermal systems unless long lasting uniform suspensions of nanoparticles are concocted. Therefore, in light of the current research’s findings, it is highly recommended to use nanoparticles of small size and low concentration. Viability of bounding surface of channels can be ensured by using hydrophobic surfaces. Moreover, further research must be conducted to evaluate the influence of thermal cycles on bounding surface and consequent influence on heat transfer rate.
- Since SiO_2_-based nanofluids display comprehensive suspension stability, it is highly recommended to concoct hybrid nanofluids containing silica as one of the two nanoparticles.

## Figures and Tables

**Table 1:** Short review of studies using silica nanofluids.

Nanoparticle	Basefluid	System	Study Type	Key Findings	Reference
Al_2_O_3_, TiO_2_ & SiO_2_	Liquid nitrogen	Plate heat exchanger	Experimental	SiO_2_-based nanofluid showed 50% less pressure-drop than rest of the nanofluids.	Javadi et al. [18]
Ag, Al_2_O_3_, Au, Cu, CuO, Fe, TiO_2_, SiO_2_, and Diamond	Water, EG, and EO	Microchannel heat sinks	Numeric	Water-based nanofluids presented optimum results. However, pressure drop for all the nanofluids was almost the same.	Kalteh [19]
Ag, Al_2_O_3_, CuO, TiO_2_, SiO_2_, and Diamond	Water	Triangular microchannel heat sinks	Numeric	Silica-based nanofluid showed the highest pressure drop and silver-based nanofluid showed the lowest pressure drop.	Mohammed et al. [20]
Al_2_O_3_, SiO_2_ and TiO_2_	Water-EG	Microchannel heat sinks	Experimental	Silicon performed much better than the basefluid and TiO_2_ in terms of heat transfer.	Rafati et al. [21]
CuO, SiO_2_, and Diamond	Water	Microchannel heat sinks	CFD Analysis	SiO_2_ nanofluid exhibited lesser thermal resistance than CuO nanofluid.	Sakanova et al. [22]
Al_2_O_3_, SiO_1_, and ZnO	Water	Shell and tube heat exchanger	Experimental	Silica nanofluid was found to be stable even by ultrasonication only.	Shahrul et al. [23]
Al_2_O_3_, CeO_2_, SiO_2_ and TiO_2_	Water	Plate heat exchanger	Experimental	Silica nanofluid outperformed the basefluid.	Tiwari et al. [24]
Fe_2_O_3_ and SiO_2_	Water	PV/T	Experimental	Electrical efficiency of the system increased by 3.051%, 3.13%, and 3.35% when cooled by water, Fe_2_O_3_, and SiO_2_ nanofluid, respectively, as compared to standalone PV modules.	Soltani et al. [25]
SiC, TiO_2_, and SiO_2_	Water	PV/T	Experimental	Electrical efficiency reached up to 11.80%, whereas, for water, it was only 11.40%.	Hasan et al. [26]
Al_2_O_3_, SiO_2_, TiO_2_, and ZnO	Water	PV/T	Experimental	5.77% increase in electrical efficiency occurred as compared to the basefluid.	Maadi et al. [27]
SiO_2_	Water	Radiator	Experimental	Silica nanofluid performed way better than the conventional coolant.	Kannan and Sivakumar [28]
SiO_2_ and TiO_2_	Water	Radiator	Experimental	Nanofluids were reported to possess great hydrodynamic as well as heat transfer potential for automotive cooling.	Hussein et al. [29]
SiO_2_	Water	Radiator	CFD Analysis	Silica nanofluid were reported to have potential to elevate the performance of radiator by up to 50% as compared to water.	Hussein et al. [30]
SiO_2_	Water	Radiator	Experimental	Topmost increment in heat transfer: 9.3%. Optimum local operation conditions: 0.4 vol.% and 60 °C.	Ebrahimi et al. [31]

**Table 2:** Detailed description SiO_2_ nanoparticles (Courtesy: Nanostructured and Amorphous Materials, Inc).

Purity (%)	99+
Approximate size (nm)	20
Morphology	Spherical
Color	White
Thermal conductivity (W/m-K)	1.4
Specific heat (j/Kg-K)	745
True density (g/cm^3^)	2.22

**Table 3:** Radiator specifications.

Radiator Geometry (mm^2^)	350 × 300, H × W
Tube Geometry (mm^3^)	350 × 1.26 × 25.13, H × W × T
Hydraulic Diameter of Tubes (mm)	75.13
Tube Area (Peripheral) (mm^2^)	574,630
Tube Area (Total) (mm^2^)	982
Perimeter (mm)	52.76
Fin Spacing (mm)	2.5
Fin Thickness (mm)	0.1

## Nomenclature

***Abbreviations***		***Greek Letters***	
A	Area (m^2^)	η	Efficiency
CHTC	Convective heat transfer coefficient (W/m^2^-K)	ρ	Density (kg/m^3^)
C_p_	Specific heat capacity (j/kg-K)	∆T	Temperature difference (K)
D_h_	Hydraulic Diameter (m)	μ	Viscosity (Pa-s)
F	Correction factor	φ	Volumetric concentration (vol.%)
H	Height (m)	***Subscripts***	
k	Thermal conductivity (W/m-K)	a	Air
L	Length (m)	bf	Base fluid
m	Mass flowrate (kg/s)	f	Liquid (water or nanofluid)
n	Shape factor	nf	Nanofluid
OHTC	Overall heat transfer coefficient (W/m^2^-K)	p	Particles
Q	Heat transfer rate (j)	r	Radiator
SEM	Scanning electron microscope	s	Surface
TEM	Transmission electron microscope	t	Tube
W	Width (m)	wf	Working fluid
***Dimensionless Numbers***			
Nu	Nusselt number		
Pr	Prandtl number		
Re	Reynolds number		
